# When Are Leaders Receptive to Voiced Creative Ideas? Joint Effects of Leaders’ Achievement Goals and Personal Sense of Power

**DOI:** 10.3389/fpsyg.2020.01527

**Published:** 2020-06-30

**Authors:** Roy B. L. Sijbom, Sharon K. Parker

**Affiliations:** ^1^Department of Work and Organizational Psychology, University of Amsterdam, Amsterdam, Netherlands; ^2^Centre for Transformative Work Design, Curtin University, Perth, WA, Australia

**Keywords:** leader receptivity, employee voice, creativity, goal orientation, power

## Abstract

Voiced suggestions for improvement and constructive change (i.e., voiced creative ideas) by employees are important for organizations. In order to reap the benefits of these ideas, leaders need to be receptive. Drawing on achievement goal theory and approach-inhibition theory of power, we examined the joint effects of leader achievement goals and personal sense of power on leader receptivity to voiced creative ideas in two studies. In a field study (Study 1, *N* = 136), we found that leaders pursuing mastery-approach goals were positively related to leader receptivity. Receptivity for leaders pursuing performance-approach goals was found to be contingent upon their personal sense of power, with a positive (negative) association under conditions of high (low) sense of power. Similarly, in experimental study (Study 2, *N* = 93), in which we manipulated leader achievement goals, the receptivity of performance-approach goal leaders was contingent upon their sense of power. When sense of personal power was high, performance-approach goal leaders displayed higher levels of receptivity than when their personal sense of power was low. An implication is that personal sense of power may prevent leaders with performance-approach goals from disregarding creative ideas that are put forward by their subordinates. These findings extend insight into how and when leaders are receptive to voiced creative ideas.

## Introduction

In light of an increasingly global, competitive, and turbulent markets, it is well recognized that employee creativity has become a key driver for organizational innovation and longer-term growth (Zhou and Shalley, 2003). Leaders realize that they can no longer succeed by merely focusing on developing own ideas (Griffin et al., 2007) and depend more than ever on employees to proactively advance bottom-up change by voicing constructive ideas for improvement. In fact, leaders acknowledge the value of employee creativity—the generation of novel and potentially useful ideas about organizational products, practices, or procedures (Amabile, 1988)—as being crucial for organizations’ future prosperity (Shalley et al., 2004). Yet, research suggests that many leaders see the voicing of creative ideas by followers as threats or distractions, and thus fail to benefit from employees’ proactive and creative voice (Detert and Burris, 2007; Sijbom et al., 2015b).

In this paper we seek to expand our theoretical understanding on how and when leaders benefit from employees’ proactive and creative voice. Specifically, we focus on leaders’ receptivity to employees’ voice of creative ideas, with receptivity defined as the degree to which leaders are willing to consider and explore creative ideas (Grant et al., 2011; Sijbom et al., 2015b). Receptivity is a necessary prerequisite for further realization of voiced creative ideas within organizations (Amabile et al., 2004; Zhou et al., 2019). Comprehending how and when leaders are receptive to these ideas is thus crucial in reaping the benefits of employee creativity. Drawing on social influence (Cialdini and Goldstein, 2004) and followership literature (Uhl-Bien et al., 2013) we view the voicing of creative ideas as a challenging proactive behavior by followers, with the attempt to influence their leaders in changing their “ways of doing things.” Leaders, however, are not passive recipients of follower influence (Oc and Bashshur, 2013) and motivational and dispositional factors amplify or attenuate their receptivity to voiced creative ideas of followers.

In fact, achievement goals have been identified as an important motivational factor at the root of leaders’ responses to voiced creative ideas (Sijbom et al., 2015a, b, 2016). We focus here on approach achievement goals rather than avoidant achievement goals as the latter have been shown to be consistently maladaptive (Payne et al., 2007). Specifically, we delve more deeply into the effects of two types of approach goals that have been considered: mastery-approach goals and performance-approach goals. Mastery-approach goals, which are centered on the development of competence, have been consistently and strongly related to positive responses. However, performance-approach goals, which are centered on the demonstration of competence, have been related to both positive and negative responses. The blend of positive and negative responses subscribes to the hybrid nature of performance-approach goals (Elliot and McGregor, 2001; Anseel et al., 2011) and makes identifying the expected relationship with voice receptivity for leaders pursuing these goals more complicated. That is, it remains unclear under what conditions leaders with performance-approach goals display more or less receptivity in response to voiced creative ideas.

To shed light on the boundary conditions influencing this relationship between performance-approach goals and receptivity, we integrate the achievement goal framework with the literature on power dynamics, which are ubiquitously present in leader-follower relations. Specifically we focus on how, with a performance-approach mindset, leaders’ receptivity might be affected by their perceptions of their abilities to influence their followers, which is referred to as leaders’ personal sense of power (Anderson et al., 2012). We focus on sense of power because, according to the approach-inhibition theory of power (Keltner et al., 2003) personal power should influence whether performance-approach leaders perceive creative input as a positive vehicle to help them perform well or perceive it as a threat to their perceived competence. Individuals who feel powerful are more approach-oriented (Galinsky et al., 2008), and will tend to perceive creative input positively. Consequently, leaders pursuing performance-approach goals should be more receptive to voice when they also have a high sense of personal power. In contrast, leaders who feel powerless are more avoidance-oriented (Galinsky et al., 2008), and so leaders with performance-approach goals will be less receptive to voice if their sense of personal power is low.

Our paper makes several theoretical and empirical contributions. First, we contribute to the body of research that focuses on the critical role that leaders fulfill in the process of translating voiced creative ideas into implemented ideas. We thus extend theorizing about the importance of motivational factors in relation to effective leadership behaviors. Leaders’ achievement goal crucially affect their receptivity and consequently (lack of) idea implementation. Second, we contribute to the literature on power by showing that possessing a sense of power prevents the situation in which leaders with performance-approach goals demonstrate ineffective leadership behaviors (i.e., disregarding creative ideas). Although leaders’ hierarchical position provides them with the power to allocate resources, their sense of power determines whether they use their position effectively. Third, our research adds to insights on leaders’ dependence on followers. Specifically, we demonstrate that leaders actively perceive and respond to follower influence. That is, leaders’ reactions to followers’ challenging proactive behaviors are shaped by leaders’ motivational and dispositional factors. To examine these relationships, we conducted two studies: a survey study of leaders working across a wide range of professions and industries and a laboratory experiment with participants who role-played the role of leader.

## Theory and Hypotheses

### Conceptualization of Achievement Goals

Achievement goals are cognitive-perceptual frameworks that describe how people define, experience, and respond to competence-relevant situations, including the workplace (DeShon and Gillespie, 2005; Elliot, 2005). Although there are various models of achievement goals, with slightly different terminology, all models make the mastery-performance distinction (Farr et al., 1993; VandeWalle, 1997; Elliot and McGregor, 2001; Payne et al., 2007). This distinction reflects differences in the definition of competence, whereby mastery goals use a self-referenced standard whereas performance goals use an other-referenced standard (Elliot and McGregor, 2001).

In dominant conceptualizations of achievement goals, mastery and performance goals are further bifurcated into approach goals, in which the focus is directed toward positive or desirable outcomes, and avoidance goals, in which the focus is on avoiding negative or undesirable outcomes (Elliot and McGregor, 2001; Baranik et al., 2010). Avoidance goals have consistently shown to be maladaptive forms of self-regulation (Payne et al., 2007). Given that adaptive rather than maladaptive behaviors are required in order to utilize the potential of voiced ideas, we focus only on approach goals because they are considered to be adaptive forms of self-regulation (Payne et al., 2007). Consequently, in our research we focus on the effects of mastery-approach and performance-approach goals (cf. Poortvliet et al., 2007; Miron-Spektor and Beenen, 2015). Mastery-approach goals entail striving to do better than before and focus on improving their own competence and exploring new knowledge or skills. Performance-approach goals entail striving to do better than others and focus on demonstrating their competence, and to seek favorable judgments from others (Elliot and McGregor, 2001; DeShon and Gillespie, 2005; Payne et al., 2007).

Achievement goals have been conceptualized as a relatively stable personality trait or as a situational domain-specific state (DeShon and Gillespie, 2005). Dispositional achievement goals refer to stable patterns of cognition and action that result from the chronic pursuit of achievement goals in different situations over time, whereas situational domain-specific achievement goals reflect a similar pattern in a specific domain (i.e., work domain) (DeShon and Gillespie, 2005; Payne et al., 2007). We follow conceptual and empirical considerations that suggest that achievement goals may be best suited for the domain-specific level (VandeWalle, 1997; Elliot, 1999; Baranik et al., 2010). Accordingly, we examined leaders’ achievement goals specific to the work domain, both assessed (Study 1) and induced (Study 2).

### Leaders’ Mastery-Approach Goals and Receptivity to Voiced Ideas

Leaders pursuing mastery-approach goals are focused on developing and gaining competence by acquiring new skills and mastering new situations (Nicholls, 1984; Dweck, 1986). Accordingly, they are likely to perceive voiced creative ideas as instrumental feedback information that provides them with important diagnostic information and suggestions for making improvements in their managerial domain. As such, voiced creative ideas represent an important source for learning for leaders that can benefit their performance and self-development. Indeed, previous findings show that, in the receipt of feedback, mastery-approach goals are positively related to outcomes that are beneficial for development and learning, such as explorative interest (Sijbom et al., 2015a), learning opportunity appraisal (Sijbom et al., 2015b) and motivation to learn (Colquitt and Simmering, 1998). In turn, these positive developmental reactions result in positive and adaptive responses to the receipt of feedback, creating a positive spiral (e.g., Sijbom et al., 2015b; Gong et al., 2017; Zhu and Akhtar, 2017). We therefore expect this earlier research to be replicated, and we propose the following:

Hypothesis 1: A higher mastery-approach goal in leaders is positively associated with leaders’ receptivity to voiced creative ideas.

### Leaders’ Performance-Approach Goals and Receptivity to Voiced Ideas

Leaders pursuing performance-approach goals have a desire to demonstrate superior leadership performance relative to others, including their followers (Nicholls, 1984; Dweck, 1986), thereby making their leader qualities an important and relevant aspect of their leadership image (Dweck and Leggett, 1988). On the one hand, leaders pursuing performance-approach goals put in effort, and persist toward these goals with the aim to outperform others. Thus, similar to leaders pursuing mastery-approach goals, voiced creative ideas might lead to effective leader behaviors because using voiced creative ideas can help leaders to fulfill their goals of outperforming others. On the other hand, performance-approach goals are associated with self-presentation concerns and fear of failure (Elliot and Church, 1997) making them vulnerable forms of regulation. Accordingly, given their focus on competence demonstration, performance-approach goal leaders may perceive followers’ creative input as evaluative feedback information that draws attention to potential deficiencies in their leadership competence, and thus threatening their desired image of being a competent leader.

These mixed associations show the somewhat complex and hybrid nature of performance-approach goals (Anseel et al., 2011). For example, performance-approach goals have been related to both positive and negative affect (Van Yperen, 2006). Also, both approach and avoidance temperament are antecedents of performance-approach goals (Elliot and Thrash, 2002), meaning that performance-approach goals potentially encompass both “approach”-related aspects and “avoidance”-related aspects. As such, other factors may be crucial to take into account because they determine whether the “positive” or “negative” aspects of performance-approach goals emerge (Anseel et al., 2011; Sijbom et al., 2015b). Accordingly we do not formulate a hypothesis concerning the direct relationship between performance-approach goals and receptivity, but instead propose the moderating role of leaders’ sense of power.

### Leaders’ Sense of Power as a Moderator

We propose that leaders’ personal sense of power may be an important leader characteristic that affects the relationship between leaders’ performance-approach goals and their receptivity to creative voice. Personal sense of power is defined as “the perception of one’s ability to influence another person or other people” (Anderson et al., 2012 p. 316). It can be viewed of as a psychological state that occurs when a person perceives that he or she is capable of influencing others. Since influence over others can be understood only in relation to others, sense of power is inherently a social-related concept (Anderson et al., 2012). In terms of the leader-follower relation, leaders’ sense of power thus relates to their feeling that they are capable of influencing their followers.

We draw on the approach-inhibition theory of power (Keltner et al., 2003) as a basis for understanding the potential moderating effect of personal sense of power on the relationship between leaders’ performance-approach goal and their receptiveness to voiced creative ideas. According to this theory, sense of power triggers the activation of the behavioral approach and inhibition systems. High levels of power activates processes associated with the behavioral approach system, such as attention to rewards and positive emotions, whereas low levels of power activates processes associated with the behavioral inhibition system, such as attention to risks or threats and negative emotions (Keltner et al., 2003). Drawing on this theoretical framework, we suggest that leaders’ sense of power acts as a crucial boundary condition that determines whether the relationship between performance-approach goals and receptivity is positive or negative. Our line of reasoning integrates the hybrid nature of performance-approach goals with the approach-inhibition theory of power.

If leaders pursuing performance-approach goals have a relative low sense of power, this activates the behavioral inhibition system. Under these conditions, the “avoidance” components of performance-approach goals are activated, meaning that leaders pay attention to risk or threats, which subsequently may inhibit them from being receptive to ideas. Creative voice challenges the current ways of doings things by signaling problems or identifying opportunities for improvement. Under conditions of low sense of power, leaders may interpret creative voice as evaluative feedback information that draws attention to potential deficiencies in their leadership competence. By perceiving voiced ideas as negative evaluative feedback regarding the self, this may cast doubt among their feelings of competence. Also, voiced ideas may be perceived as being threatening to their desired image of being a competent leader. By voicing creative input, subordinates may highlight that some state of affairs that are under the leaders’ responsibility for overseeing are insufficient or at least suboptimal. Leaders may thus worry about appearing incompetent in the eyes of others because the voiced creative ideas may signal inferiority of their leadership competence rather than the superiority they aim for. Due to this image threat appraisal, performance-approach goal leaders can be expected to become motivated to preserve their image, thereby inhibiting them from being receptive to creative voice.

In contrast, relative high levels of sense of power triggers the activation of the behavior approach system. Under these conditions, performance-approach goal leaders are more focused on rewards and positive outcomes. Thus, high sense of power can be expected to activate the “approach” component of performance-approach goals, leading them to pay more attention to the instrumental value of the creative voice rather than possible (negative) social consequences. Creative voice is instrumental because it may help leaders reaching their goal of appearing competent and gaining favorable (competence) judgments. Previous research from the feedback domain showed when the “approach” component is activated, performance-approach goals pay more attention to the content of the feedback (Anseel et al., 2011). Also, Sijbom et al. (2015b) showed that performance-approach goal leaders may be receptive to voiced ideas when employees do not highlight underlying problems. Accordingly, under conditions of high sense of power, leaders may show effective leadership behaviors (i.e., relative high levels of receptivity to voiced ideas). We test the following hypothesis:

Hypothesis 2: Leaders’ sense of power moderates the association between leaders’ performance-approach goals and their receptiveness to voiced creative ideas, such that this association is positive for leaders with a high sense of power, and negative when leaders’ sense of power is low.

Mastery-approach goal leaders are less concerned with influencing other people. Rather they are focused on developing their own competences and skills as a leader. Owing to this learning interest, mastery-approach goal leaders can be expected to be receptive to voiced creative ideas, irrespective of their sense of power, and hence we focus only on main effects for this variable.

## Overview of Studies

**FIGURE 1:**
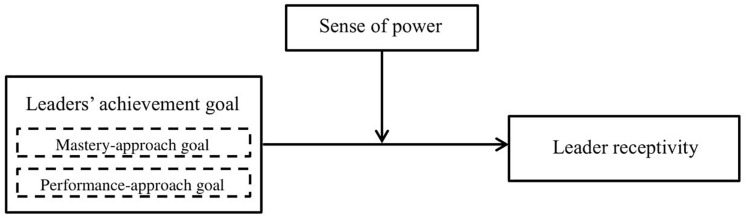
Research model.

Our research model is depicted in Figure 1. We tested our hypotheses in two studies using different methodologies (field study and experimental study) and different samples (leaders and students). In Study 1, we measured our variables in a survey in a sample of leaders. In Study 2, we manipulated achievement goals in a sample of students who role-played the role of leader and performed a management marketing task. Study 1 thus has the advantage of showing evidence of the phenomenon in real work settings, albeit being weak in terms of disentangling causality, whereas Study 2 has the advantage of demonstrating causal effects of achievement goals on receptivity, albeit suffers from low generalizability.

## Study 1

### Method

#### Sample and Procedure

We recruited a total of 137 participants from Amazon’s Mechanical Turk (MTurk) to complete an online questionnaire in exchange for $1,50. In order to get a relevant sample, a system qualification was used such that only individuals located in the United States could participate. Furthermore, respondents had to explicitly answer a question (“yes” or “no”) whether they held a supervisory position with at least three subordinates. Only when they answered “yes” they could proceed with the survey. Finally, at the end of the survey respondents indicated how many years they were in a supervisory position and how many subordinates they supervised. With these questions we checked whether the respondents met our inclusion criteria. One respondent was excluded because he/she indicated to not supervise any subordinates, leaving *N* = 136 (81 male, *M*_age_ = 34.5 years, *SD*_age_ = 10.9). The respondents’ mean total work experience was 15.2 years (*SD* = 9.8); mean total work experience in a supervisory position was 7.1 years (*SD* = 6.8); and mean number of subordinates supervised was 10.3 (*SD* = 14.9; with a minimum of 3 and a maximum of 156).

The questionnaire first assessed participants’ general sense of power and their achievement goals. They were then asked to think about a situation in which a follower voices a creative idea, after which their receptivity toward that idea was assessed.

#### Measures

Leaders’ performance-approach goal (α = 0.93) and leaders’ mastery-approach goal (α = 0.79) were measured using the corresponding three-item subscales of the Achievement Goal Questionnaire-Revisited (AGQ-R; Elliot et al., 2011). Items were adapted to fit the work context of the research by changing the domain from a class setting (“In my classes”) to a work setting (“In my work”; for similar adaptations see Sijbom et al., 2015b; Sijbom et al., 2019). Participants rated three items for the performance-approach goal construct (e.g., “My aim is to outperform other colleagues in my work”) and three items for the mastery-approach goal construct (e.g., “My aim is to perform better in my work than I have done in the past”). Response categories ranged from 1 (*not true*) to 7 (*extremely true*).

Personal sense of power (α = 0.87) was assessed using the eight-item scale developed and validated by Anderson and Galinsky (2006). Items were adapted to fit the work context by including “at work” in each item (Anderson et al., 2012). Sample items include “In my relationships with others at work I can get people to listen to what I say” and “In my relationships with others at work my ideas and opinions are often ignored (reverse-coded).” Response categories ranged from 1 (*strongly disagree*) to 7 (*strongly agree*).

Leaders’ receptivity (α = 0.66) was measured using four items. Leaders were first asked to think about a situation in which a subordinate voices a creative idea. Then they answered the four items to assess their receptivity. Two items were based on Sijbom et al. (2015b): “How likely is it that you would like to discuss the ideas together with the subordinate?” and “How likely is it that you would let the subordinate know that you will work out the creative idea together?”. Two other items were created for the purpose of this study: “How likely is it that you thank the subordinate for thinking along, but will ignore the creative idea?” (reverse-scored) and “To what extent do you want to show support for the creative idea?”. The response categories ranged from 1 (*not at all*) to 7 (*very much*).

We investigated total work experience (in years) as a potential control variable. Leaders who have more years of work experience have shown to be positively related to adoption decisions (Damanpour and Schneider, 2006), which may confound the examined relationships. We also included power sharing as a potential control variable using a five-item scale (α = 0.75) from De Hoogh and Den Hartog (2008); example item is “I allow subordinates to influence critical decisions”). Leaders who share their power with their subordinates can also be expected to more receptive to ideas and suggestions of these subordinates, which may confound the examined relationship in this research. Finally, performance-avoidance goal (α = 0.93; e.g., “My aim is to avoid doing worse than other colleagues in my work”) and mastery-avoidance goal (α = 0.87; e.g., “My goal in my work is to avoid doing worse than I have done before”) were measured as potential control variables using the corresponding three-item subscales of the AGQ-R (Elliot et al., 2011). Research shows that both avoidance goals are correlated with both approach goals (Payne et al., 2007).

Of these potential control variables, only tenure and power sharing were significantly correlated with one (or more) of the independent variables and the dependent variable (see Table 1) and were included as control variables in our analyses (Becker, 2005; Becker et al., 2016).

**TABLE 1:** Means, standard deviations, and correlations (Study 1).

Variable	Mean	SD	1	2	3	4	5	6	7	8
1. Gender^a^	–	–								
2. Tenure^b^	15.23	9.79	–0.02							
3. Power sharing	4.96	0.94	–0.15	0.06						
4. Mastery-avoidance goal	4.84	1.64	0.18*	−0.22*	0.08					
5. Performance-avoidance goal	4.88	1.66	0.22*	−0.23*	–0.08	0.54**				
6. Mastery-approach goal	5.91	0.98	0.22*	0.01	0.17*	0.36**	0.10			
7. Performance-approach goal	5.24	1.55	0.08	−0.19*	–0.14	0.22*	0.62**	0.07		
8. Personal sense of power	5.53	0.92	0.15	0.17*	0.07	–0.05	–0.02	0.25*	0.08	
9. Receptivity	5.16	0.98	0.11	0.20*	0.44**	0.08	0.11	0.30**	0.02	0.32**

N = 136. ^*^p < 0.05; ^**^p < 0.001. ^a^0 = “male”, 1 = “female”. ^b^N = 135.

### Results

Table 1 displays the means, standard deviations, and correlations of the variables included in our study.

Table 2 displays the results the hierarchical regression analyses. After standardizing the independent variables (Aiken and West, 1991) we entered them into the regression analysis in three consecutive steps. In the first step, the control variables (tenure and power sharing) were entered. In the second step the main effects variables of mastery-approach goals, performance-approach goals, and personal sense of power were entered. In the third step, the interaction terms were entered.

**TABLE 2:** Results of regression analyses.

	Receptivity				
Steps and variables	Model 1	Model 2	Model 3	Model 4	Model 5
**Step 1 (control variables)**					
Tenure	0.17*	0.15*	0.16*	0.17*	0.17*
Power sharing	0.43**	0.39**	0.39**	0.40**	0.40**
**Step 2 (independent variables)**					
Mastery-approach goal		0.18*	0.17*	0.19*	0.19*
Performance-approach goal		0.08	0.08	–0.00	0.00
Sense of power		0.23**	0.23**	0.24**	0.24**
**Step 3 (interaction terms)**					
Mastery-approach goal × sense of power			0.07		0.03
Performance-approach goal × sense of power				0.22**	0.21**
Δ*R*^2^	0.22**	0.11**	0.00^a^	0.04^a^*	0.04^a^*
Adjusted *R*^2^	0.21**	0.31**	0.30**	0.34**	0.34**

N = 136. Standardized regression coefficients are reported for the respective regression steps. ^a^ΔR^2^ represents the incremental variance explained over Model 2. *p < 0.05; **p < 0.001.

Hypothesis 1 stated that higher mastery-approach goals in leaders would be positively associated with their receptivity toward voiced ideas. As can be seen in Model 2, a significant effect of leader mastery-approach goal on receptivity was found, *b* = 0.17, *SE*_*b*_ = 0.08, β = 0.18, *p* = 0.022, thereby providing support for Hypothesis 1. Also, Model 2 shows that personal sense of power had a significant positive effect on leader receptivity, *b* = 0.22, *SE*_*b*_ = 0.08, β = 0.23, *p* = 0.004. Although we did not formally hypothesize this relationship, this finding was expected based on the literature showing that sense of power activates approach behaviors.

The main effect of performance-approach goal on receptivity was not significant, *b* = 0.08, *SE*_*b*_ = 0.07, β = 0.08, *p* = 0.310. However, as stated in Hypothesis 2, we expected leaders’ sense of power to moderate the association between leaders’ performance-approach goals and their receptiveness to voiced creative ideas, such that this association is positive for leaders with a high sense of power, and negative when leaders’ sense of power is low. The coefficient associated with the performance-approach goal × personal sense of power interaction term was significant (Model 5; *b* = 0.19, *SE*_*b*_ = 0.07, β = 0.21, *p* = 0.008) and this interaction explained incremental variance in leader receptivity beyond main effects (see Model 4), Δ*R*^2^ = 0.04, *F*(1, 127) = 8.01, *p* = 0.005. As recommended by Becker et al. (2016) we also tested a model without the control variables. The results for the main effects remain similar. The coefficient associated with the performance-approach goal × personal sense of power interaction term became marginally significant (*b* = 0.15, *SE*_*b*_ = 0.08, β = 0.16, *p* = 0.065). However, the performance-approach goal × personal sense of power interaction still explained incremental variance in leader receptivity beyond main effects (Δ*R*^2^ = 0.03, *F*(1, 131) = 4.08, *p* = 0.045. These changes in results with and without control variables indicate that the control variables do affect the outcomes and are relevant to include. We therefore proceeded with the results that include control variables.

**FIGURE 2:**
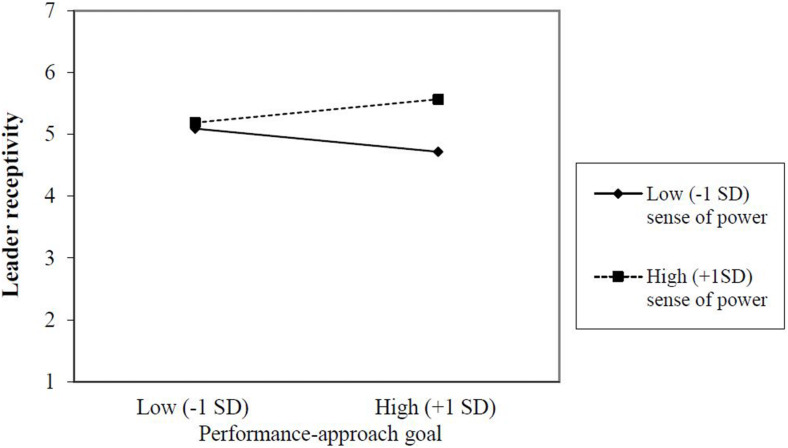
Interaction effect of leaders’ performance-approach goal and sense of power on leader receptivity.

Based on inspection of the interaction plot (see Figure 2), we can conclude that the association between leaders’ performance-approach goal and receptivity differs significantly in the hypothesized direction at different levels (one standard deviation above the mean score and one standard deviation below the mean score) of personal sense of power. We conducted simple slope analyses to further interpret our significant interaction (Aiken and West, 1991; Dawson, 2013). Results showed a significant positive association between leaders’ performance-approach goals and receptivity under conditions of high (+1 SD) personal sense of power, *b* = 0.19, *SE*_*b*_ = 0.08, b = 0.19, *p* = 0.024, and a non-significant negative association under conditions of low (−1 SD) personal sense of power, *b* = −0.19, *SE*_*b*_ = 0.12, β = −0.19, *p* = 0.130. These results indicate that under conditions of high personal sense of power, the individual slope does significantly differ from 0. Accordingly, we found partial support for Hypothesis 2. A relevant supplementary question is whether receptivity differs for leaders high on performance-approach goals. Results showed a significant positive association between sense of power and receptivity for leaders high on performance-approach goals (+1 SD), *b* = 0.43, *SE*_*b*_ = 0.10, b = 0.43, *p* < 0.001, and a non-significant association under conditions for leaders low on performance-approach goals (−1 SD), *b* = 0.05, *SE*_*b*_ = 0.10, β = 0.05, *p* = 0.626. These results indicate that leaders high on performance-approach goals are sensitive to sense of power, with higher (lower) levels of receptivity when sense of power is high (low).

## Study 2

Study 2 concerns an experimental study in which leader achievement goal (performance-approach goal vs. mastery-approach goal) is manipulated rather than measured, thereby enabling us to more strongly show that achievement goals cause different levels of receptivity. Therefore, we reformulated Hypothesis 2 into a testable form for Study 2. As above, we do not expect receptivity of leaders with induced mastery-approach goals to be affected by leaders’ sense of power.

Hypothesis 2: Leaders with induced performance-approach goals, rather than leaders with induced mastery-approach goals, display higher (lower) levels of receptivity under conditions of high (low) sense of power.

### Method

#### Participants and Design

A total of 98 Australian business school undergraduates participated in an online experimental study for partial course credit. Personal sense of power was assessed in a survey prior to participation in an experiment. Participants were randomly assigned to one of the two conditions (performance-approach goal condition vs. mastery-approach goal condition) of the between-subjects design. Five participants were excluded as they did not provide a short narrative as part of the achievement goal manipulation, leaving a final sample of *N* = 93 [of whom 50,5% were female; *M*_age_ = 19.59, *SD*_age_ = 1.7; performance-approach goal condition (*n* = 44); mastery-approach goal condition (*n* = 49)]. Gender and age had no effects and are not discussed further.

#### Procedure

After signing informed consent, participants completed the general sense of power questionnaire. Next, participants performed a management marketing task (for details, see Sijbom et al., 2015a). In this task, participants were assigned a leadership role and performed an in-basket task in which they had to respond to emails from their subordinates. Specifically, participants were assigned to the role of the company’s marketing manager, who was responsible for positioning and selling fast-food products on the market. In the scenario, the organization had developed a new product, so-called fat-free fries, and a project team was composed to successfully introduce the product to the market. Besides the marketing manager, who operated as the team leader, the project team consisted of three subordinates. The marketing manager assigned the team members the task of developing informative sentences that could be used as input for crafting the final marketing strategy propagated by the marketing manager. In actuality, the team members were nonexistent, and in their role of marketing manager, the participants received standardized input. After responding to the input of two team members, the participants received an e-mail from a third team member, named Sandy. In the e-mail, Sandy proposed the use of a different marketing strategy to introduce the new product, which prior research has shown that it is judged as being a creative (i.e., novel and potential useful) marketing strategy in the context of the company (Sijbom et al., 2015a). Given that Sandy communicated this creative idea for renewing the marketing strategy to the leader, Sandy’s proposal can be considered to be a voiced creative idea. After completing the dependent variables and the manipulation checks, the participants were debriefed and thanked for their participation.

#### Achievement Goal Manipulation

To manipulate the achievement goal of participants, we used the achievement goal manipulation procedure developed and previously used by Sijbom et al. (2015a; 2015a; 2015b). The manipulation consisted of three coherent aspects from which a specific achievement goal was derived. First, different information with respect to the organizational climate (competitive vs developmental climate) was given. Second, a personal leadership motto was imposed on the participants. The motto in the performance-approach goal condition was: “Managers are superiors and, therefore, must demonstrate their superior competences in their executive work with subordinates.” In the mastery-approach condition, the motto was: “Managers are developers and, therefore, must keep developing their competences in their executive work.” The participants then had to write a short narrative in which they clearly advocated their characteristic leadership motto and had to describe their emotions and beliefs associated with it. This narrative procedure is used to intensify the manipulation (Poortvliet et al., 2007). Finally, participants were given a specific goal that varied according to condition. Participants in the performance-approach goal condition read the following: *“In line with your motto, your goal as a leader is to demonstrate your leadership competences to your subordinates*.*”* In the mastery-approach goal condition participants read the following: *“In line with your motto, your goal as a leader is to develop your leadership competences”* (Sijbom et al., 2015a).

### Measure

#### Achievement Goal Manipulation Check

Participants had to indicate which leadership motto they held as a manager. Participants could choose between (1) “Managers are superiors and, therefore, must demonstrate their superior competences in their executive work with other” (performance-approach goal condition), (2) “Managers are developers and, therefore, must keep developing their competences in their executive work” (mastery-approach goal condition), and (3) “I did not receive information with respect to a motto.”

Also, we assessed the degree to which participants were committed to their assigned achievement goal. We used a five-item scale to assess goal commitment (α = 0.78; Klein et al., 2001). After participants had their specific achievement goal assigned, they answered the following items: “I am strongly committed to pursuing this goal”; “It wouldn’t take much to make me abandon this goal” (reverse-coded); “I think this is a good goal to shoot for”; “It’s hard to take this goal seriously” (reverse-coded); and “Quite frankly, I don’t care if I achieve this goal or not” (reverse-coded). The response categories ranged from 1 (*strongly disagree*) to 7 (*strongly agree*).

Personal sense of power (α = 0.88) was assessed using the same eight-item scale of Anderson and Galinsky (2006) as in Study 1. The correlation with the dummy-variable of achievement goal condition was non-significant (*r* = −0.06, *p* = 0.570).

Leader receptivity (α = 0.77) was assessed using a four-item scale. All items started with the stem: “How likely is it that you will let Sandy know that…”, followed by different statements: “…you would like to discuss the input together with Sandy?; “…you seriously want to discuss the input during the next meeting of the project team?”; “…you want to further develop the input together with Sandy?”; and “…you will not use Sandy’s input” (reverse-coded). The first three items were developed and used by Sijbom et al. (2015b). We developed the fourth negatively framed item for the purpose of this study. The response categories ranged from 1 (*extremely unlikely*) to 7 (*extremely likely*).

### Results

#### Manipulation Checks

In the performance-approach goal condition, 73% answered the information check correctly. This was 94% in the mastery-approach goal condition. Results of a *t*-test revealed that the goal commitment scores of participants in the performance-approach goal condition (*M* = 5.47, *SD* = 1.00) were not significantly different from the goal commitment scores of participants in the mastery-approach goal condition (*M* = 5.60, *SD* = 0.87), *t*(91) = −0.68, *p* = 0.50. This indicates that participants in both conditions were committed to their recommended goal. Hence, the manipulation of achievement goals was successful. When participants who incorrectly answered this question were excluded, the pattern of results was the same and still significant.

#### Leader Receptivity

To examine the interactive effect of achievement goal and personal sense of power on leader receptivity, we performed a regression analysis. Achievement goal (0 = performance-approach goal condition, 1 = mastery-approach goal condition), standardized personal sense of power, and their interaction were used as the independent variables. The analysis revealed a nonsignificant main effect of achievement goal, *B* = −0.03, *SE*_b_ = 0.18, β = −0.02, *p* = 0.86, 95% CI for B [−0.40, 0.33], meaning that leaders with an induced performance-approach goal did not statistically differ from leaders with in induced mastery-approach goal with respect to their receptivity. Although not formally hypothesized, a significant main effect of personal sense of power was found, *B* = 0.26, *SE*_b_ = 0.09, β = 0.29, *p* = 0.006, 95% CI for B [0.08, 0.44].

**FIGURE 3:**
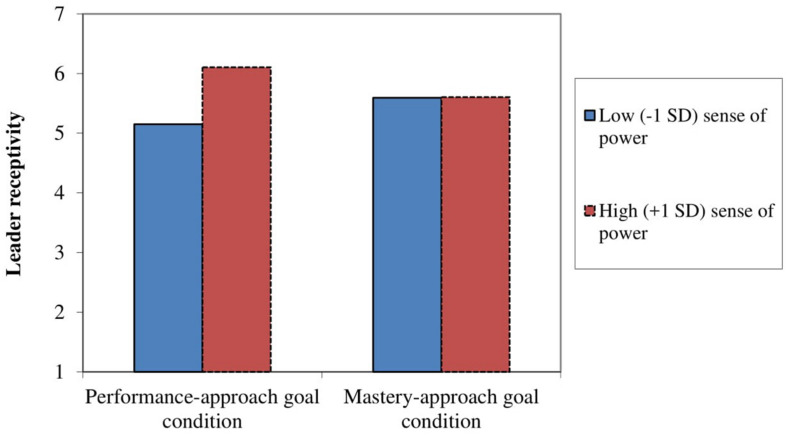
Interaction effect of achievement goals (0 = performance-approach goal condition; +1 = master-approach goal condition) and sense of power on leader receptivity.

Hypothesis 2 predicted an interaction between leaders’ achievement goals and sense of power such that leaders with induced performance-approach goals, rather than leaders with induced mastery-approach goals, display higher (lower) levels of receptivity under conditions of high (low) sense of power. The analysis revealed a significant interaction between achievement goal and personal sense of power (see Figure 3), *B* = −0.47, *SE*_b_ = 0.18, β = −0.36, *p* = 0.01, 95% CI for B [−0.83, −0.11]. Simple slope analyses showed that receptivity of leaders in the mastery-approach goal condition did not vary at different levels of sense of power, *B* = 0.02, *SE*_b_ = 0.13, β = 0.02, *p* = 0.88, 95% CI for *B* [−0.24, 0.27]. For leaders in the performance-approach goal condition, leader receptivity did significantly vary at different levels of sense of power, with higher (lower) levels of leader receptivity under conditions of high (low) sense of power, *B* = 0.49, *SE*_b_ = 0.13, β = 0.54, *p* < 0.001, 95% CI for *B* [0.24, 0.74]. Together these results provide support for Hypothesis 2 that the receptivity of performance-approach goal leaders is moderated by their sense of power.

## Discussion

Being receptive to creative voice is crucial for leaders to benefit from employee creativity. In the present research, we investigated how and when leaders show effective leader behaviors, that is, when they are receptive in response to voiced creative ideas. Building on achievement goal theory and approach-inhibition theory of power, we showed in two studies that receptivity of leaders pursuing performance-approach goals is contingent upon their sense of power. That is, leaders pursuing performance-approach goals were more receptive when they had relatively high levels of sense of power, and were less receptive when they had relatively low levels of sense of power. Furthermore, leaders pursuing mastery-approach goals were associated with more receptivity toward voiced creative ideas. This relationship was not contingent upon their sense of power. Together, these studies confirm our basic notion that in their reactions toward voiced ideas, leaders pursuing performance-approach goals are sensitive to their sense of power.

### Theoretical Implications

Our study contributes to the literature investigating the receiving side of creativity. Rather than identifying and investigating antecedents and determinants of voice (Chamberlin et al., 2017) we answer to recent calls in the literature to focus more on the receiving side of voiced creativity (Zhou et al., 2019). Our study adds to the importance that perceiver characteristics have on their reactions toward voiced creative ideas.

First, our study showed that achievement goals of leaders are an important motivational factor that affects their reactions to voiced creative ideas, with mastery-approach goals being clearly positively related to leaders’ receptivity. This finding is in line with earlier research showing that leaders pursuing mastery-approach goals were positively related to the adoption of voiced creative ideas (Sijbom et al., 2015a). As such, these results add to the literature on the role of motivational factors in relation to creative voice endorsement (Zhou et al., 2019). Also, these results add to the robustness of the idea that mastery-approach goals are related to adaptive responses to proactive behaviors of employees.

Second, and related to the important role of leader achievement goals, our study sheds light on the hybrid nature of performance-approach goals (Elliot and Church, 1997; Anseel et al., 2011). Importantly, we identified sense of power, being a perceiver characteristic, as a crucial boundary condition that can clarify when pursuit of performance-approach goals might results in (in)effective leader behaviors. When leaders in pursuit of performance-approach goals have a high sense of power, they show adaptive behaviors toward voiced ideas, whereas they show maladaptive behaviors when experiences low sense of power. These results underscore the importance of perceiver characteristics (Zhou et al., 2017) and as such help to unravel and better understand the hybrid nature and responses of leaders pursuing performance-approach goals. Our study expands earlier studies that have identified moderators of the performance-approach goal-outcome relationship, including type of feedback (Anseel et al., 2011) characteristics of the creative idea (Sijbom et al., 2015b) and characteristics of the creative idea sender (Sijbom et al., 2015a, 2016). Importantly, our study focuses on attributes of the leader as key influencers on the social process of receptivity, rather than this earlier research that has focused mostly on attributes of the idea or the voicer. Focusing on leader attributes mean our research identifies important implications for leader development, as we discuss shortly.

Finally, we provide implications for the literature on the psychology of power. Specifically, we show that for those with structural power (i.e., leaders), sense of power is a relevant characteristics to take into account (see also, Fast et al., 2012; Haselhuhn et al., 2017). While leaders have formal power over their employees, their sense of power varies which affects their endorsement, such as receptivity to voiced ideas. Our study shows that sense of power operates in two ways. One way sense of power operates relates to direct positive effects on receptivity. This is in line with earlier findings showing that leaders with a high sense of power seize more opportunities than those with low sense of power (Sturm and Antonakis, 2015). The other way in which sense of power operates is in terms of moderating the effects of the relationship between performance-approach goals and receptivity. That is, high sense of power can enable leaders pursuing performance-approach goals to overcome the tendency to show maladaptive responses when receiving ideas from their employees. Altogether, our findings suggest that sense of power is an important variable for leader receptivity.

### Practical Implications

Organizations that want their leaders to be more receptive to voiced creative ideas, should stimulate mastery-approach goals among leaders. One way organizations may realize this is by creating a working environment in which leaders are stimulated to develop skills and competences. To achieve this, organizations should aim to install and establish specific practices aimed at learning. For instance, by emphasizing evaluation in terms of progress and effort, by defining success in terms of development and improvement and by accepting mistakes as part of the learning process, organizations may be able to create such a learning-focused working environment (Ames, 1992; Dragoni, 2005).

Receptivity to voiced ideas may also be enhanced by increasing a sense of power among leaders, especially when they have strong performance-approach goals. Organizations can help leaders in this regard by cultivating their sense of power. First, leaders can activate their sense of power by recalling an experience in which they had power or felt powerful. Second, research demonstrated that several individual differences are associated with personal sense of power. For example, individuals who focus more on the positive, rewarding aspects of themselves and their relationships (behavioral approach system) have a higher sense of power than those who attend to more negative, and threatening aspects of their relationships (behavioral inhibition system) (Anderson et al., 2012). Also, internal locus of control is positively associated with sense of power (Anderson et al., 2012). If organizations want leaders with relatively high levels of sense of power, they may focus on such individual differences in recruitment and selection processes. Finally, our findings also suggest that if organizations make effort to reduce leaders’ feelings of low power, this may help to make them more receptive. Since sense of power is about individuals’ perceived capability to influence others, organizations should consider designing interventions aimed at techniques that have been shown to increase self-efficacy, such as role modeling (learning from other leaders), verbal persuasion, and enactive mastery (Bandura, 1997). For example, one way to enhance feelings of power may be through coaching (Edmondson, 2003) with coaching being a form of verbal persuasion. Also higher-level management might enhance feelings of power among leaders through structural empowerment (Seibert et al., 2004; Spreitzer, 1995) such as through work redesign to increase leaders’ job autonomy (Parker, 2014).

### Limitations and Directions for Future Research

Our study has several desirable features (e.g., different methodologies, different samples, and different operationalization of achievement goals). In Study 1 we measured our variables in a survey study among a sample of leaders, which is good for external validity. At the same time, Study 1 used a cross-sectional design, which does not allow for making causal inferences. In Study 2, we used an experimental design, that allows us to demonstrate causal effects of achievement goals on receptivity, albeit suffers from low generalizability.

Besides these strengths, our studies have some limitations. First, in our studies we focused on leader reactions to creative voice. Therefore, in our studies we used an idea that was creative (both novel and useful). A limitation is that it is not possible to test whether different levels of creativity of the voiced idea are influencing the results (Zhou et al., 2019). Future research may therefore investigate leaders’ responses to voiced creative and uncreative ideas.

Second, in Study 1 we relied on an MTurk sample. Although we checked whether participants met our study inclusion criteria (i.e., holding a managerial position), we did not include attention checks nor did we screen for HIT completion success rates (Chmielewski and Kucker, 2020) which limits us in determining the quality of the data.

Third, in Study 2 73% of participants in the performance-approach goal condition indicated the correct motto (that is, passed the manipulation check). This percentage is lower compared to previous studies using the same manipulation (Sijbom et al., 2015a, b) and may explain why we did not found any differences in receptivity between the mastery-approach goal condition and the performance-approach goal condition^1^.

Fourth, we investigated leader receptivity to voiced ideas. Although, receptivity is an important first step of idea endorsement, further steps need to be taken to implement voiced creative ideas (Burris, 2012; Li et al., 2019). These steps include getting more detailed information to further validate the idea, evaluating the pros and cons, and considering whether the idea is feasible in terms of available resources. Future research may therefore include measures that also capture the idea implementation part. Additionally, since we only used self-report measures for receptivity, future research may include more objective measures of receptivity and endorsement, like allocation of resources (i.e., budgets).

A final limitation has to do with our focus on investigating sense of power as a moderator of the relationship between achievement goals and receptivity. As a result, our studies did not investigate underlying mechanisms that can explain why the interaction between leaders’ performance-approach goals and sense of power leads to differences in leader receptivity. Therefore, future research may investigate process mechanisms such as image threat appraisal (Sijbom et al., 2015a) and effort in processing the idea (Li et al., 2019) that may explain the moderating effects we found in the present research.

## Conclusion

In this paper we examine leader receptivity as being an important outcome in order to reap the benefits of voiced creative ideas. Across two studies, using different methodologies and samples, we found that achievement goals of leaders determine their receptivity. Mastery-approach goals are positively related to receptivity. For performance-approach goals their effects where contingent upon their personal sense of power. That is, leaders pursuing performance-approach goals were more receptive when they had relative high levels of sense of power, and were less receptive when they had relatively low levels of sense of power. All in all, the results underscore the importance of achievement goals leaders pursue on their ability to reap the benefits of voiced creative ideas.

## Data Availability Statement

The datasets generated for this study are available on request to the corresponding author.

## Ethics Statement

The studies were reviewed and approved by the Ethical Committee of the University of Groningen (Study 1) and the Human Research Ethics Office of the University of Western Australia (Study 2). Online informed consent was obtained from all participants.

## Author Contributions

RS contributed to the theorizing, data collection, data analysis, interpretations of results, and writing. SP contributed to the theorizing, interpretations of results, and writing. RS and SP are collectively responsible for the final completion of the manuscript. All authors contributed to the article and approved the submitted version.

## Conflict of Interest

The authors declare that the research was conducted in the absence of any commercial or financial relationships that could be construed as a potential conflict of interest.

## Footnotes

1 Please note that a study of Sijbom et al. (2016) Study 2 that investigated integrative management of creative ideas, also did not find significant differences between the mastery-approach goal condition and the performance-approach goal condition. Future research is therefore needed to better understand the (lack of) differential effects of achievement goals.
